# Romance Scams: Romantic Imagery and Transcranial Direct Current Stimulation

**DOI:** 10.3389/fpsyt.2021.738874

**Published:** 2021-10-11

**Authors:** Jie-Yu Chuang

**Affiliations:** ^1^Department of Psychiatry, Cardinal Tien Hospital, New Taipei City, Taiwan; ^2^School of Medicine, College of Medicine, Fu Jen Catholic University, New Taipei City, Taiwan

**Keywords:** romantic relationship, imagery, romance scam, visual network, suicide, tDCS

## Abstract

Love has an enormous effect on mental health. One does not need an actual romantic relationship to be in love. Indeed, romantic love can be built upon without frequent or real-life encounters, such as with a stranger from a matching website. With the advancement of the Internet and the influence of coronavirus disease, it is believed that these distant romantic relationships and related romance scams are burgeoning. Often, the victims of scams keep emotionally attached to the scammer even after the lie is revealed, which is hypothesized to be attributed to the aberrantly exaggerated romantic imagery of the victims. It is observed that many victims suffer from symptoms similar to a post-traumatic stress disorder, and some even consider suicide. However, there is scant literature on this topic. In this article, it is further postulated that the aberrant romantic imagery might be associated with impulsive acts such as suicide once the ideal but fake romantic relationship is dissolved. Thereafter, it is further speculated that manipulation of the visual network, possibly by transcranial direct current stimulation (tDCS), might be a promising treatment.

## Romance Scams

Romance scams are virtual relationships generally constructed through websites for deceiving victims to extort money from them (1). In 2008, in the United States, victims of online dating fraud lost an average of 3,000 dollars (1). According to US Federal Trade Commission, reported losses to romance scams reached a record $304 million in 2020, possibly related to the pandemic (2). Moreover, many victims exhibit symptoms similar to those of post-traumatic stress disorder long after the event, with some even contemplating suicide (3). However, this issue has seldom been addressed in the current literature.

The criminal often uses fake identity (stage 1: the profile), grooms the victim (stage 2: grooming), and intensely communicates with the victim over months or even years. Once the victim falls in love with the criminal, various excuses are made to steal money from the victim (stage 3: the sting) (4). Romance scam victims tend to be middle-aged, well-educated women with impulsive and addictive disposition (5). In a qualitative study, many victims described the scammers as “ideal partners” and perceived the relationships are therapeutic in which they feel free to disclose every aspect of themselves (3). Indeed, most victims found the loss of the relationship more upsetting than financial loss (3). Most victims in the study found it challenging to separate the criminal from the fake identity, even after they were told that the criminal was of a different race or sex (3). In the study, even after seeing the real picture of the scammer, one victim continued to experience strong affection toward the scammer, and she could not visualize that someone other than the person in the profile was writing messages to her (3). She described her feelings: “I'd had this guy's picture in my house for a year…I mean your feelings don't go away, it doesn't work like that at all… I saw him writing those things…those words came from him, they didn't come from…” (3). It appears that the victim kept visualizing (or imaging) her fake lover for a long period; thus, making it challenging to discard the imagination despite efforts. As described in the following sections, it is speculated that the rationale supporting this entanglement of fake and reality is aberrantly exaggerated romantic imagery.

## Mental Imagery and Romantic Imagery

Conscious cognition can occur in mental imagery or verbal-linguistic formats (6). Mental imagery, also known as the mind's eye, is an amazing ability to create a conscious sensory experience at will, allowing us to travel through space and time and enjoy our virtual worlds (7) in an as-if-real manner, as if it were a weak form of perception (8). Mental imagery (e.g., an image of flowers) has been shown to evoke greater emotional impact than verbal-linguistic representations (e.g., depicting flowers as the seed-bearing part of a plant) of both positive and negative information (6). It serves to maintain and amplify emotional states with downstream impacts on motivation and behavior (6). It can involve all senses; however, visual imagery is the focus of this study. Since humans are visual creatures with a vast proportion of cortical tissues assigned to process visual information compared to the other senses, visual imagery is assumed to be the principal player in mental imagery (7).

Aberrancy in mental imagery has been found ubiquitously in mental disorders. For instance, exaggeratedly vivid imagery of the abused substance is associated with anxiety, craving, and subsequent relapse in substance-related disorders (6); patients with obsessive-compulsive disorder might have mental images of bacteria over their hands evoking anxiety and later fueling repeated washing (8); depressed patients may suffer from misfortunate future images or “flash-forwards” suicidal images (8, 9). Post-traumatic stress disorder (PTSD) is also characterized by intrusive mental imagery of trauma (7).

Romantic imagery is defined as the mental imagery of an object of romantic love. It is postulated that a much more intense romantic imagery is needed to maintain an online romantic relationship than a conventional one when partners have more actual contact. Indeed, it has been argued that it is imagination that enables long-distance romantic relationships to be continuous beyond face-to-face meetings and intimacy to be experienced without co-presence (10). Based on a qualitative analysis of fraudulent profiles from an international dating website, Kopp et al. suggest that romantic imagery of the victims play a critical role in romance scams (11). Namely, the scammers often pretend to be the ideal partners fitting into the romantic imagery of the victims (11). There are large individual differences in mental imagery ability, ranging from aphantasia to hyperphantasia (7). Using revised NEO Personality Inventory, it has been shown that aphantasia is related to reduced extraversion whereas hyperphantaisa is linked to increased openness (12). Correspondingly, individual differences in mental imagery ability might be associated with social competence. Presumably, this inter-individual variation also exists in romantic imagery. Online relationships may work better in individuals with greater romantic imagery.

Mental imagery has been shown to facilitate subsequent perceptions. Studies using the “binocular rivalry” technique have demonstrated that when participants visualize one of two patterns, the imaged pattern is perceptually dominant in a subsequent brief single binocular rivalry presentation (8). Consequently, it is assumed that if one mainly imaged the merits of a romantic object, one could hardly see or feel his/her demerits, particularly in a distant romantic relationship, when people tend to hide their downsides effortlessly. Moreover, victims of romance scams tend to idealize their fake relationships (1). This idealization might elucidate the puzzling fact that romance scam victims are well-educated (5).

The enormous power of romantic imagery could perhaps be best depicted by the love between the famous Italian poet Dante Alighieri (1265 to 1321) and his beloved Beatrice Portinari. It is believed that Dante had only met Beatrice twice briefly, yet she became his muse, true love, salvation, and guide in “Heaven in Divine Comedy” (13). Currently, with the advent of the Internet and the influence of coronavirus disease, online romantic relationships are burgeoning. A systematic review showed that high scores on the romantic belief of idealization are associated with the likelihood of being an online romance scam victim (1). In addition to romance scams, these distant online relationships could face rising problems like “ghosting,” a way of ending a relationship suddenly by stopping all communication without an explanation (14). Since victims of “ghosting” might never meet their persecutors in person, in addition to the fact that their romantic imagery is destroyed, they are also left without an opportunity to ask for a reason. They might suffer from social pain, distress, loneliness, and depression (14). Moreover, serious relationship dissatisfaction has been shown to predict a significant risk for major depressive disorders (15). Impulsive acts such as suicide or homicide may be associated with the ending of romantic imagery (16). Despite these grave consequences, most victims never seek clinical help or meet the diagnostic criteria of major psychiatric disorders [some of them may be diagnosed with relationship problems according to the Diagnostic and Statistical Manual of Mental Disorders, Fifth Edition (15)].

## Neural Mechanism of Romantic Imagery

Contrary to the bottom-up pathway of normal perception, a top-down general model of voluntary mental imagery involves the following steps: beginning in the frontal cortex, retrieving memories from medial temporal areas (such as the hippocampus), and forming sensory representations from the parietal areas and the visual system (such as the fusiform face area and visual cortex V1) (7). Accordingly, based on measurements from 464 participants, a recent meta-analysis indicated activation in the left superior parietal lobule, inferior premotor areas, inferior frontal sulcus, supplementary and cingulate eye fields, frontal eye fields, parahippocampal region, fusiform gyrus, and primary visual cortex (V1) during mental imagery (17). People with more vivid mental imagery ability tend to have a larger frontal cortex and smaller primary visual cortex (V1 and V2) (7).

There are a limited number of studies exploring the neural mechanism of romantic love, with most studies recruiting a small number of participants. A series of functional magnetic resonance imaging (fMRI) studies conducted by Fisher et al. concluded that the reward system, in particular, the ventral tegmental area, striatum, and caudate, show enhanced activation when participants (whether they are in the early stage of love, long-term love, or rejected) view a photograph of their beloved ones as compared to photographs of an acquaintance (18–21). However, I argue that romantic imagery might barely be captured in this series of studies as participants focus on real-time external photograph stimuli briefly instead of immersing in their inner mental imagery. Two studies were found to better explore the neural mechanisms of romantic imagery. In the first fMRI study, nine women whose romantic relationships ended within the preceding 4 months showed increased activation in the posterior brain regions (cerebellum, occipital cortex, and posterior temporoparietal regions) and decreased activation in the anterior brain regions (striatum, anterior cingulate, and prefrontal cortex) when ruminating about their loved ones, compared to their neutral thoughts about a different person (22). In the second fMRI resting-state study, compared with people who have never been in love, those who were intensely in love showed a decreased connectivity degree in the orbitofrontal cortex and increased connectivity degree in the fusiform gyrus (23). Based on the results of these two studies, it is speculated that similar to mental imagery, the frontal cortex and visual network play important roles in romantic imagery.

## Proposed Treatment for Romance Scams

Romance scam victims suffer from shame, embarrassment, shock, anger, anxiety, stress, fear, depression, or even suicidal ideation (3). Furthermore, romance scam victims might experience symptoms resembling major depressive disorder, griefing, or post-traumatic disorder (PTSD) (3). Due to embarrassment or fear of disapproval, many victims did not disclose the romance scams to close others or seek external support (3). Nevertheless, it is advised that romance scam victims should seek professional help once significant functional impairment is noted (e.g., self-harm ideation or inability to work). Since the aberrantly exaggerated romantic imagery is hypothesized to be the core element in romance scams, it might be more efficient and effective to manipulate the primary romantic imagery itself than to treat a myriad of secondary symptoms (e.g., depression, anxiety, or sleep problems). Hopefully, manipulation of the romantic imagery will be followed by mitigation of psychological impact of romance scams.

Abnormalities in mental imagery have been implicated in many mental disorders. Since mental imagery can exacerbate the effects of interpretation bias in mental disorders, manipulation of the mental imagery might alleviate mental symptoms (24). Various techniques have been implemented in clinical settings to manipulate mental imagery. These techniques can be roughly grouped into three categories: one is to change the content of mental imagery (imagery re-scripting, elicitation of competing for mental imagery, and positive imagery training); the second is to desensitize mental imagery by repeated exposure (systematic desensitization); and the third is to introduce distraction and interfere with the formation of mental imagery (eye movement desensitization and reprocessing, EMDR) (6, 8). For example, negative outcome image (e.g., performing badly) is replaced by positive image (e.g., performing competently) during the practice of “imagery re-scripting” in social phobia (8, 25). In one version of “positive imagery training,” participants are instructed to generate positive mental imagery when they listen to brief descriptions of everyday situations (initially ambiguous but always resolve positively) (26). During the practice of “systematic desensitization,” gradual exposure to mental images of feared objects (e.g., spiders) might be paired with physical relaxation (8, 27). The practice of “eye movement desensitization and reprocessing” (EMDR) appears to dampen the vividness of mental imagery by promoting lateral eye movements during the recall of emotional memories (8, 28). Some novel techniques have been developed to reduce the emotional impact of mental imagery by introducing concurrent task interference. For instance, participants who view a traumatic film clip experience fewer mental imagery intrusions and less distress after playing a visuospatial computer game (Tetris) (29). It has been theorized that playing Tetris occupies the visual areas of the brain and impairs one's ability to replay the traumatic scene during memory consolidation (7). Other techniques to introduce concurrent task interference and reduce mental imagery include: making hand movements, watching a dynamic visual noise array, or constructing shapes from modeling clay (30).

Although romantic imagery research is still in its infancy, logically, it is possible to manipulate romantic imagery borrowing techniques from mental imagery intervention. Nevertheless, contrary to the most intrusive and unwanted nature of mental imagery in mental disorders, romantic imagery often has a soothing effect, providing hope, and enjoyment. Consequently, romantic imagery might be more resilient and challenging to alter than mental imagery. Therefore, a more powerful treatment modality might be needed to manipulate romantic imagery in refractory cases.

Keogh et al. demonstrated that a stronger mental imagery ability is associated with a lower visual cortex excitability and higher prefrontal cortex excitability. They assumed that hyperexcitability of the visual cortex acts as a source of noise and limits the sensitivity of the neural response to top-down mental imagery signals from the prefrontal cortex, resulting in weaker mental imagery, whereas hyperexcitability of the prefrontal cortex amplifies the top-down signals and boosts mental imagery. Non-invasive transcranial direct current stimulation (tDCS) is a tool to modulate neurons by inducing a subthreshold shift of resting membrane potentials toward depolarization or hyperpolarization. Transcranial direct current stimulation does not trigger action potentials, but most likely affects the spike timing of individual neurons such that anodal stimulation is usually followed by depolarization of neurons whereas cathodal stimulation is usually followed by hyperpolarization of neurons (31). Recent evidence suggests that tDCS can provide substantial treatment benefits on various mental disorders: major depressive disorder (32); bipolar depression (33); schizophrenia spectrum disorders with improvements in positive, negative, and cognitive symptoms (34); and borderline personality disorder with improvements in executive functions, cognitive control, and emotional regulation (35).

Home usage of tDCS might be available in the future owing to its easy management and low cost of devices (36). In people suffering from PTSD, mental imagery is associated with re-experiencing sub-symptom (7). Utilizing 2 mA tDCS in the dorsolateral prefrontal cortex, a greater reduction in re-experiencing sub-symptom has been found in PTSD patients (37). In another study, utilizing 1.5 mA tDCS, Keogh et al. were able to change the mental imagery ability by manipulating the excitability of the visual cortex and the prefrontal cortex, with the former showing a more significant effect. However, they have suggested that more complex mental imagery, such as imagery of faces, may rely less on the early visual cortex and more so on the excitability further upstream, such as the fusiform face area (38). Indeed, a recent neuroimaging meta-analysis indicates that fusiform gyrus might play a more dominant role than early visual cortex in mental imagery (39). Consequently, tDCS stimulation of the visual network (fusiform face area) might mitigate the exaggerated romantic imagery in refractory cases.

Last but not least, if suicidal ideation is manifested in romantic scam victims, a more thorough diagnostic investigation (e.g., Structured Clinical Interview for DSM-5) and prompt evidence-based intervention (e.g., pharmacologic treatment, cognitive behavioral therapy, or problem-solving therapy) (40) is warranted.

## Discussion

In conclusion, romantic imagery with the activation of the visual network is hypothesized to be more extensively utilized in romance scams than conventional romantic relationships. Traditional techniques should be used to manipulate romantic imagery. In refractory cases, the intervention of the visual network (tDCS of the fusiform face area) could be a promising treatment for this aberrant romantic imagery in victims who suffer from romance scams. Future studies are warranted to test this hypothesis.

Limitations of this hypothesis include: the dearth of previous literature on romance scams intervention and the lack of actual empirical evidence to prove this hypothesis. Effectiveness of this hypothesis should be tested in the real-world clinical practice in the future.

Transcranial direct current stimulation is generally regarded as a safe and tolerable technique modulating neural activity painlessly and non-invasively with weak electrical currents (41). There is no evidence for irreversible injury produced by conventional tDCS protocals (≤40 min, ≤4 mA, ≤7.2 Coulombs) (42). Nevertheless, some tDCS-related adverse events have been reported, such as treatment-emergent mania/hypomania (TEM) and skin lesions similar to burns (41). Despite the scant literature, a meta-analysis indicates that there is no significant association between TEM and tDCS (43). In a recent paper, Pilloni et al. suggest that risk of skin lesions can be prevented by appropriate methodological rigor (e.g., single-use saline-soaked sponges with reliable headset for the placement of electrodes are preferred) (44). However, more tDCS research is warranted in vulnerable populations such as pregnant women, children, the elderly, patients with epilepsy, stroke, or mood disorders (42).

Victims of romance scams face “double hits” (financial loss and emotional trauma). Moreover, many of them are too embarrassed to seek help or confess to their family or friends (3). People often react to encounters with disbelief and disdain. “Why on earth would you believe this guy who you never met?” However, people do not understand that romance scams are delicately well-planned frauds implemented by professional scammers who spend months or even years on the victims. These victims continue to vividly imagine their fake lovers for a long time, and it is challenging to destroy their affection just by perceiving that they have been deceived. Consequently, more research attention should be paid to the intervention of romance scams.

## Data Availability Statement

The original contributions presented in the study are included in the article/supplementary material, further inquiries can be directed to the corresponding author/s.

## Author Contributions

The author confirms being the sole contributor of this work and has approved it for publication.

## Funding

J-YC is funded by Cardinal Tien Hospital.

## Conflict of Interest

The author declares that the research was conducted in the absence of any commercial or financial relationships that could be construed as a potential conflict of interest.

## Publisher's Note

All claims expressed in this article are solely those of the authors and do not necessarily represent those of their affiliated organizations, or those of the publisher, the editors and the reviewers. Any product that may be evaluated in this article, or claim that may be made by its manufacturer, is not guaranteed or endorsed by the publisher.
